# Melatonin rescues cardiovascular dysfunction during hypoxic development in the chick embryo

**DOI:** 10.1111/jpi.12283

**Published:** 2015-10-26

**Authors:** Nozomi Itani, Katie L. Skeffington, Christian Beck, Youguo Niu, Dino A. Giussani

**Affiliations:** ^1^ Department of Physiology, Development and NeuroscienceUniversity of CambridgeCambridgeUK

**Keywords:** antioxidant, cardiovascular disease, melatonin, prevention of fetal programming

## Abstract

There is a search for rescue therapy against fetal origins of cardiovascular disease in pregnancy complicated by chronic fetal hypoxia, particularly following clinical diagnosis of fetal growth restriction (FGR). Melatonin protects the placenta in adverse pregnancy; however, whether melatonin protects the fetal heart and vasculature in hypoxic pregnancy independent of effects on the placenta is unknown. Whether melatonin can rescue fetal cardiovascular dysfunction when treatment commences following FGR diagnosis is also unknown. We isolated the effects of melatonin on the developing cardiovascular system of the chick embryo during hypoxic incubation. We tested the hypothesis that melatonin directly protects the fetal cardiovascular system in adverse development and that it can rescue dysfunction following FGR diagnosis. Chick embryos were incubated under normoxia or hypoxia (14% O_2_) from day 1 ± melatonin treatment (1 mg/kg/day) from day 13 of incubation (term ~21 days). Melatonin in hypoxic chick embryos rescued cardiac systolic dysfunction, impaired cardiac contractility and relaxability, increased cardiac sympathetic dominance, and endothelial dysfunction in peripheral circulations. The mechanisms involved included reduced oxidative stress, enhanced antioxidant capacity and restored vascular endothelial growth factor expression, and NO bioavailability. Melatonin treatment of the chick embryo starting at day 13 of incubation, equivalent to *ca*. 25 wk of gestation in human pregnancy, rescues early origins of cardiovascular dysfunction during hypoxic development. Melatonin may be a suitable antioxidant candidate for translation to human therapy to protect the fetal cardiovascular system in adverse pregnancy.

## Introduction

In addition to the interaction between genetic makeup and traditional lifestyles risk factors, such as smoking, obesity, or a sedentary life, it is now established that a component of heart disease‐risk can be predetermined by adverse conditions before birth during early development 1, 2. A common consequence of adverse pregnancy is chronic fetal hypoxia, and several independent studies in humans and animal models have reported that chronic fetal hypoxia can trigger a fetal origin of cardiac dysfunction and increase the risk of cardiovascular disease in later life 3, 4, 5, 6, 7, 8, 9, 10. Therefore, there is accumulating interest in establishing the mechanisms underlying this association to identify potential therapeutic strategies for intervention.

Previously, our laboratory raised the hypothesis that oxidative stress may be a candidate mechanism linking chronic fetal hypoxia, a fetal origin of cardiovascular dysfunction and programming of cardiovascular disease in the adult offspring 8. Chronic fetal hypoxia promotes oxidative stress in the fetal cardiovascular system 4, 5, 8, 9 and maternal treatment with the antioxidant vitamin C in hypoxic pregnancy prevented the programming of cardiovascular dysfunction in the adult offspring 8. Although the latter study provided proof of principle to support the hypothesis tested, only high doses of vitamin C incompatible with human treatment were effective to act as an antioxidant in vivo. Further, both maternal treatment with vitamin C and fetal hypoxia started concurrently in early gestation. In human clinical practice, intrauterine growth restriction (IUGR) as a result of chronic fetal hypoxia may be only reliably diagnosed at around 25 wk of gestation. Therefore, there is interest in identifying alternative translational antioxidant therapy in tolerable doses that may rescue cardiovascular dysfunction triggered by chronic fetal hypoxia once diagnosis has been feasible.

An alternative candidate therapeutic agent is melatonin. Melatonin is a powerful antioxidant that increases nitric oxide (NO) bioavailability because of its combined ability to directly scavenge free radicals, to enhance endogenous antioxidant enzyme capacity and to reduce the leakage of electrons from the mitochondrial electron transport chain through improving its function. In addition, the metabolites of melatonin also possess antioxidant properties. Such antioxidant roles of melatonin have been comprehensively and extensively reviewed in recent reports 11, 12. Further, melatonin treatment protects placental perfusion, fetal growth, and fetal cardiovascular function in pregnancy complicated by chronic adverse intrauterine conditions 13, 14, 15. Consequently, a human clinical trial testing the efficacy to protect fetal growth of maternal oral melatonin administration in complicated pregnancy has recently been launched 16. However, whether the antioxidant actions of melatonin protect the developing fetal heart and circulation directly, independent of its protective effects on the placenta and umbilical perfusion in adverse pregnancy is completely unknown.

The chick embryo is the only animal model that permits isolation of the effects of hypoxia and of antioxidant therapy on fetal growth and on the developing cardiovascular system independent of effects on the placental and/or maternal physiology 17, 18, 19, 20. Therefore, in this study, we have tested the hypothesis that melatonin has direct protective effects on the fetal heart as well as the fetal vasculature in adverse development. Adopting an integrative approach at the isolated organ, cellular, and molecular levels, we investigated the effects of melatonin treatment in chick embryos incubated under normoxic or hypoxic conditions from day 1 of development on cardiac function, peripheral vascular reactivity, cardiac stereology and vascular morphology, and on cardiac molecular indices of oxidative stress, antioxidant capacity, and of NO bioavailability. Treatment of chick embryos with melatonin started at day 13 of incubation, which is equivalent to *ca*. 25 wk of gestation in human pregnancy.

## Methods

### Animals

All procedures were performed under the UK Animals (Scientific Procedures) Act 1986 and were approved by the Ethical Review Committee of the University of Cambridge. Fertilized Bovans Brown eggs (Medeggs, Norfolk, UK) were weighed and incubated under normoxic (21% O_2_) or hypoxic (14 ± 0.5% O_2_) conditions (37.9°C, 45% humidity, 12:12 hr light:dark cycle, automatic rotation every hour, Masalles incubator Mod‐75A, equipped with electronic servo‐controlled humidity cool steam injection system HS‐Auto‐3.5L; Masalles, Barcelona, Spain) from day 1. The levels of oxygen, humidity, and temperature inside the incubators were continuously monitored (DD103 DrDAQ Oxygen Sensor, Pico Technology, St. Neots, UK). Chick embryos were treated with melatonin (1 mg/kg/day, Sigma‐Aldrich, Dorset, UK) or vehicle (100 *μ*L water for injection, Norbrook Laboratories, Corby, UK) from day 13 to day 18 of incubation. Melatonin was injected daily into the air cell onto the chorioallantoic membrane via a 1‐mm hole in the eggshell. The hole was covered with sticky tape at all other times. All treatment procedures were performed under sterile conditions. The dose of melatonin was extrapolated from experiments in our laboratory, which showed successful antioxidant effects in rodent pregnancy 13.

### Analysis of hematocrit and growth

On day 19, embryos underwent euthanasia by cervical spinal transaction and their body weight was recorded. Blood was collected in micro‐hematocrit tubes (Vitrex, Modulohm, Denmark) directly from the heart in duplicate, and the hematocrit was determined. The heart and brain were dissected and weighed. The heart was snap frozen in liquid nitrogen and stored at −80°C until molecular analysis.

### Molecular analysis of oxidative stress, antioxidant defences, and vascular endothelial growth factor (VEGF) in the embryo heart

The expression of 3‐nitrotyrosine (3‐NT), 4‐hydroxynonenal (4‐HNE) and superoxide dismutase (SOD), the activity of catalase, and the levels of total nitrate and nitrite (NOx) in the 19‐day chick embryo heart were determined by commercial assay kits (3‐NT: ab116691, Abcam, Cambridge, UK, 4‐HNE: E12H0203, AMS biotechnology, Abington, UK, SOD: Sigma‐Aldrich, Catalase: 707002; Cayman Chemical Company, Ann Arbor, MI, USA, NOx: 78001, Cayman Chemical Company).

The expression of glutathione peroxidase (GPx) and of VEGF protein in the 19‐day chick embryo heart was determined by Western blot, as described 21. Heart protein (10 *μ*g) was loaded onto each lane, and Coomassie blue staining was used as a loading control. GPx (anti‐GPx 1, Abcam) and VEGF (anti‐VEGF, Abcam) primary antibodies and HRP‐conjugated anti‐rabbit secondary antibody were used. Proteins were detected using West Pico Chemiluminescent substrate (Thermo Scientific, Loughborough, UK). The membranes were visualized by exposing them to a photosensitive film (GE Healthcare Amersham Hyperfilm ECL, Fisher Scientific, Loughborough, UK). The intensities of the bands were analyzed with the AlphaEase imaging software (Alpha Innotech, San Leandro, CA, USA).

### Analysis of cardiac function using the Langendorff preparation

In another group of embryos, the heart was rapidly excised following cervical transection on day 19 of incubation. The heart was immediately placed in ice‐cold Krebs–Henseleit Buffer (KHB, NaCl:120 mm, KCl:4.7 mm, MgSO_2_.7H_2_O:1.2 mm, KH_2_PO_4_:1.2 mm, NaHCO_3_:25 mm, glucose:10 mm, CaCl_2_.2H_2_O:1.3 mm) and mounted onto the apparatus. The heart was perfused through the coronary arteries via the aorta with KHB (40°C, gassed with 95% O_2_ and 5% CO_2_) at a constant pressure (40 cm H_2_O). A small incision was made in the left atrium, and a small balloon was inserted into the left ventricle via the atrium. The balloon was filled with distilled water using a 100‐*μ*L Hamilton syringe and attached to a rigid distilled water‐filled catheter connected to a calibrated pressure transducer (Argon Medical Devices, Plano, TX, USA). The balloon volume was adjusted to 30 *μ*L to obtain a value for left ventricular end diastolic pressure (LVEDP) between 5 and 10 mmHg 8, 22, 23. After the stabilization period, baseline recordings were made for heart rate (HR), LVEDP, and left ventricular systolic pressure (LVSP). Left ventricular developed pressure (LVDP) was calculated by subtracting LVEDP from LVSP. The maximum first derivative (d*P*/d*t*_max_) and the minimum first derivative (d*P*/d*t*_min_) of the left ventricular pressure were calculated using an IDEEQ data acquisition system (version 0‐2.5.0, Maastricht, Netherlands). Coronary flow rate (CFR) was determined by timed collections of cardiac perfusate effluent.

Changes in HR and LVDP in response to the sympathetic *β*_1_ adrenoreceptor agonist isoprenaline ((−)‐Isoproterenol (+)‐bitartrate salt, Sigma‐Aldrich, 10^−9^–10^−7^ m) and to the parasympathetic muscarinic agonist carbachol (carbamylcholine chloride, Sigma‐Aldrich, 10^−8^–10^−6^ m) were measured. The HR and LVDP responses were expressed as a percentage change from the baseline, and then, the ratio of the maximal HR (chronotropic) and LVDP (inotropic) responses to isoprenaline and to carbachol was calculated. A recovery time ranging between 5 and 15 min was allowed between each treatment bolus to permit HR and LVDP to stabilize at baseline values before the administration of the next bolus.

### Cardiac stereology and morphology of the aorta

On day 19 of incubation, another group of embryos were anaesthetized with sodium pentobarbital (0.1 mL i.p. Pentoject; Animalcare Ltd, York, UK). The heart was exposed, an incision was made in the right atrium, and it was perfusion fixed at a constant pressure of 2.66 kPa 24 with 10% formaldehyde via a needle inserted into the left ventricle. A segment of the fixed descending aorta at the level of the cardiac apex and the fixed heart were stored in formaldehyde for 24 hr and then in phosphate buffered saline at 4°C until further analysis. The heart was sectioned at 1 mm thickness with a heart slicer (Zivic Instruments, Pittsburgh, PA, USA), and the sections were imaged on a light box. Quantitative analysis of the heart was performed using ImageJ (version 1.46, National Institute of Health,Bethesda, MD, USA) by superimposing a point grid on the cardiac sections. The volumes of the left ventricle (LV), left lumen, right ventricle (RV), and right lumen were quantified using the Cavalieri's principle 25. The segments of aorta were embedded in a paraffin block and then sectioned at 5 *μ*m thickness. Ten consecutive sections were collected from the distal end and stained with hematoxylin and eosin. Quantitative analysis was performed using the Computer‐Assisted Stereology Toolbox system (CAST version2.0, Olympus, Albertslund, Denmark).

### Analysis of peripheral vascular reactivity using wire myography

A third‐order branch of the left femoral artery was dissected under a dissecting microscope (Stemi 2000; Zeiss, Cambridge, UK) from the same embryos used for the analysis of cardiac function via the Langendorff preparation at day 19 of incubation. Arterial segments (2 mm) were threaded with two pieces of stainless steel wire (40 *μ*m diameter) and secured in a 4‐chamber microvascular myograph (Multi Wire Myograph System 610M; DMT, Aarhus, Denmark). The chamber contained 5 mL of cold Kreb's buffer (NaCl:118.5 mm, KCl:4.75 mm, MgSO_4_.7H_2_0:1.2 mm, KH_2_PO_4_:1.2 mm, NaHCO_3_:25.0 mm, CaCl_2_:2.5 mm, glucose:5.55 mm, gassed with 95% O_2_ and 5% CO_2_) and it was heated slowly to 37°C. The segment was equilibrated for 20 min and then stretched until a physiological transmural pressure of 2.66 kPa was achieved (DMT normalization module; DMT). Vasodilator response to cumulative doses of sodium nitroprusside (SNP, 10^−10^–10^−4^ m) and of acetylcholine (ACh, 10^−9^–10^−5^ m) was assessed after preconstricting the vessel with a suboptimal dose of potassium (K^+^, <85% of maximal response to K^+^ 125 mm). The partial contributions of endogenous NO‐dependent and NO‐independent mechanisms to the vasorelaxation were determined by repeating the ACh dose–response curve after incubating the vessel with L‐NAME (10^−5^ m, 10 min). Vascular constrictor capacity was assessed with increasing doses of K^+^ solutions (16.74–250 mm) and of phenylephrine (PE, 10^−8^–10^−4^ m). The response to K^+^ was normalized to the diameter of the vessel (mN/mm/*μ*m/1000). The response to phenylephrine was normalized to the response to 125 mm K^+^ of the same vessel (%K^+^125). LabChart was used for data acquisition and analysis (Labchart 6.0, Powerlab 8/30; AD Instruments, Chalgrove, UK).

### Analysis of circulating melatonin concentration

To estimate the likely diurnal concentration of circulating melatonin concentration achieved in the chick embryo during daily dosing with melatonin, another nine groups of normoxic chick embryos (n = 3–6 per group) and another nine groups of hypoxic chick embryos (n = 3–6 per group) were administered 1 mg/kg on day 18–19 of incubation as before and blood was collected at 0, 0.5, 1, 2, 3, 4, 6, 8, or 24 hr following treatment. The embryos were killed by cervical transection, and blood was collected directly from the heart into dipotassium EDTA tubes (Teklab, Durham, UK) and centrifuged for 5 min at 2370 rcf. Supernatant was stored at –80°C until analysis. The plasma melatonin concentration was determined using a commercial ELISA kit (CSB‐E08132h, CUSABIO, Wuhan, China). The detection range of the assay was 3.13–800 pg/mL. The intra‐assay coefficient of variation (CV) was <8%, and the interassay CV was <10%.

### Statistical analysis

All data are expressed as mean ± S.E.M. Statistical comparisons were made using one‐ or two‐way ANOVA with the Tukey or Bonferroni post hoc test, as appropriate. For all comparisons, statistical significance was accepted when *P* < 0.05 (Graphpad prism version 5.00, Graphpad Software, Inc. San Diego, CA, USA).

## Results

**Figure 1:**
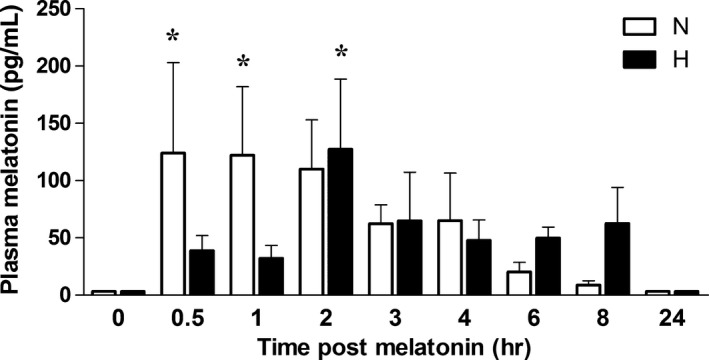
Plasma melatonin concentration in normoxic and hypoxic chick embryos with or without melatonin treatment. Values are mean ± S.E.M. of plasma melatonin levels in day 19 chick embryos incubated in normoxia or hypoxia before (0 min) or after (0.5, 1, 2, 3, 4, 6, 8, or 24 hr) a bolus dose of melatonin (5 *μ*g). Groups are normoxia (N, white histogram, n = 36) and hypoxia (H, black histogram, n = 39). Significant (*P* < 0.05) differences are as follows: * versus 0 hr of the corresponding group (Two‐way ANOVA with Bonferroni post hoc test).

In normoxic embryos, plasma melatonin concentrations peaked at 124 ± 78 pg/mL between 0.5 and 1 hr following treatment, after which melatonin decreased progressively toward basal levels (Fig. 1). In contrast, in hypoxic embryos, although plasma melatonin concentrations peaked at similar concentrations (127 ± 61 pg/mL), this elevation occurred at 2 hr following treatment, after which melatonin decreased more irregularly toward basal levels (Fig. 1). However, there was no difference in the total circulating concentration of melatonin achieved within the 24‐hr day in normoxic (517 ± 253, n = 36) or hypoxic (402 ± 176 pg/mL, n = 39) embryos.

**Figure 2:**
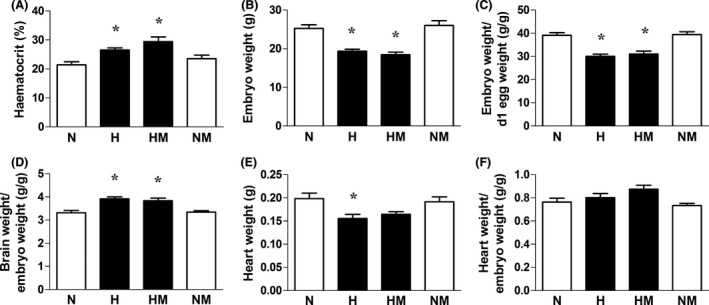
Hematocrit and fetal biometry in normoxic and hypoxic chick embryos with or without melatonin treatment at day 19 of incubation. Values are mean ± S.E.M. at day 19 of hematocrit (A), absolute embryo weight (B), relative embryo weight (C), brain weight relative to body weight (D), heart weight (E), and heart weight relative to body weight (F) of chick embryos incubated in either normoxia (N, n = 10), hypoxia (H, n = 10), hypoxia with melatonin (HM, n = 11), or normoxia with melatonin (NM, n = 10). Significant (*P* < 0.05) differences are as follows: * versus N (one‐ or two‐way ANOVA with Tukey and Bonferroni post hoc test, respectively).

Relative to normoxic embryos, embryos incubated under hypoxic conditions showed significantly elevated hematocrit, reduced absolute body weight, or body weight expressed relative to the initial egg mass, an enhanced brain‐to‐body weight ratio and decreased absolute heart weight in proportion to the reduced body weight (Fig. 2A–F). Melatonin treatment in hypoxic embryos did not affect the enhanced hematocrit, reduced body weight, or increased brain‐to‐body weight ratio; however, the absolute heart weight was no longer significantly different from normoxic embryos (Fig. 2A–F). Melatonin treatment in normoxic embryos did not affect any of these variables (Fig. 2A–F).

**Figure 3:**
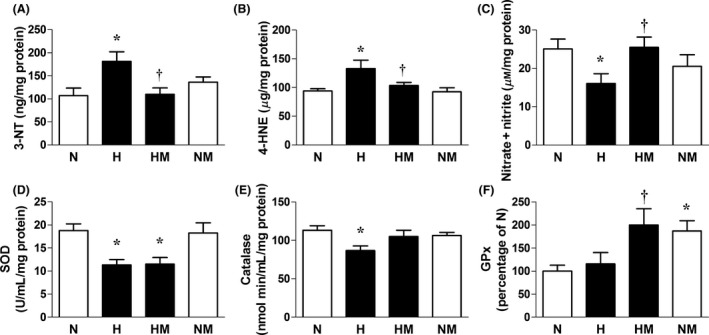
Indices of oxidative stress and antioxidant defences in hearts from normoxic or hypoxic chick embryos with or without melatonin treatment at day 19 of incubation. Values are mean ± S.E.M. at day 19 of the expression of 3‐nitrotyrosine (A), the expression of 4‐hydroxynonenal (B), total nitrate and nitrite concentration (C), the expression of superoxide dismutase (D), catalase activity (E), and the expression of glutathione peroxidase (F) in the hearts of chick embryos incubated in either normoxia (N, n = 9), hypoxia (H, n = 10), hypoxia with melatonin (HM, n = 10), or normoxia with melatonin (NM, n = 10). n = 6 for all groups for (F). Significant (*P* < 0.05) differences are as follows: * versus N. † H versus HM (one‐ or two‐way ANOVA with Tukey and Bonferroni post hoc test, respectively).

**Figure 4:**
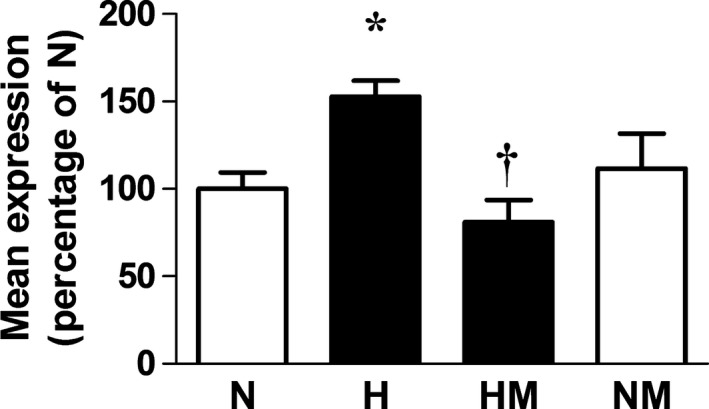
Cardiac vascular endothelial growth factor (VEGF) expression in hearts from normoxic or hypoxic chick embryos with or without melatonin treatment at day 19 of incubation. Values are mean ± S.E.M. at day 19 of the heart VEGF expression in the chick embryo incubated in either normoxia (N, n = 6), hypoxia (H, n = 6), hypoxia with melatonin (HM, n = 6), or normoxia with melatonin (NM, n = 6). Significant (*P* < 0.05) differences are as follows: * versus N. † H versus HM (one‐ or two‐way ANOVA with Tukey and Bonferroni post hoc test, respectively).

Relative to normoxic embryos, hearts of embryos incubated under hypoxic conditions showed significantly enhanced expression of 3‐NT and 4‐HNE but significantly decreased levels of combined nitrate and nitrite concentrations. Hearts of embryos incubated under hypoxic conditions also showed a significantly reduced expression of SOD and significantly impaired catalase activity but no change in GPx protein expression (Fig. 3D–F). Melatonin treatment in hypoxic embryos prevented the increase in cardiac 3‐NT and 4‐HNE levels and restored cardiac NOx levels (Fig. 3A–C). Melatonin treatment in hypoxic embryos also augmented the cardiac catalase activity, and it significantly enhanced the cardiac GPx expression but it did not affect the impaired cardiac expression of SOD (Fig. 3D–F). Melatonin treatment in normoxic embryos also led to a significant increase in the cardiac GPx expression (Fig. 3F). Relative to normoxic embryos, hearts of embryos incubated under hypoxic conditions showed significantly enhanced expression of VEGF (Fig. 4). Melatonin treatment in hypoxic embryos restored the cardiac VEGF expression to control values. Melatonin treatment in normoxic embryos did not affect the cardiac expression of VEGF (Fig. 4).

**Figure 5:**
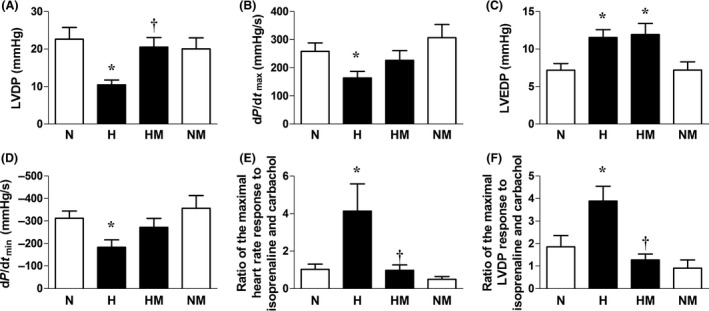
Cardiac function in normoxic or hypoxic chick embryos with or without melatonin treatment at day 19 of incubation. Values are mean ± S.E.M. at day 19 of the left ventricular developed pressure (LVDP, A), myocardial contractility (d*P*/d*t*_max_, B), left ventricular end diastolic pressure (C), myocardial relaxability (d*P*/d*t*_min_, D), the ratio of the maximal heart rate responses to isoprenaline and to carbachol (chronotropic sympathetic dominance, E), and the ratio of the maximal LVDP response to isoprenaline and to carbachol (inotropic sympathetic dominance, F) of chick embryos incubated in either normoxia (N, n = 10), hypoxia (H, n = 10), hypoxia with melatonin (HM, n* =* 11), or normoxia with melatonin (NM, n = 10). Significant (*P* < 0.05) differences are as follows: * versus N. † H versus HM (one‐ or two‐way ANOVA with Tukey and Bonferroni post hoc test, respectively).

Relative to normoxic embryos, embryos incubated under hypoxic conditions showed significantly impaired LVDP, d*P*/d*t*_max_, and d*P*/d*t*_min_, but significantly elevated LVEDP and an enhanced value for the ratio of the maximal HR (chronotropic) and LVDP (inotropic) response to isoprenaline and carbachol (Fig. 5A–F). Melatonin treatment in hypoxic embryos restored LVDP, d*P*/d*t*_max_, d*P*/d*t*_min_, and the ratios of the chronotropic and inotropic responses to isoprenaline and carbachol. However, LVEDP remained significantly elevated compared to normoxic embryos. In addition, while hypoxic incubation alone did not affect CFR, melatonin treatment in hypoxic embryos significantly increased CFR relative to normoxic embryos (N:6.7 ± 0.4, H:9.3 ± 0.9, HM:13.5 ± 1.9*, NM:9.2 ± 1.2 mL/min/g of heart tissue, *P* < 0.05; * versus N). Melatonin treatment in normoxic embryos did not affect cardiac function. The ratio of the maximal LVDP and HR responses to isoprenaline and carbachol was not different in normoxic embryos with or without melatonin treatment (Fig. 5E,F).

**Figure 6:**
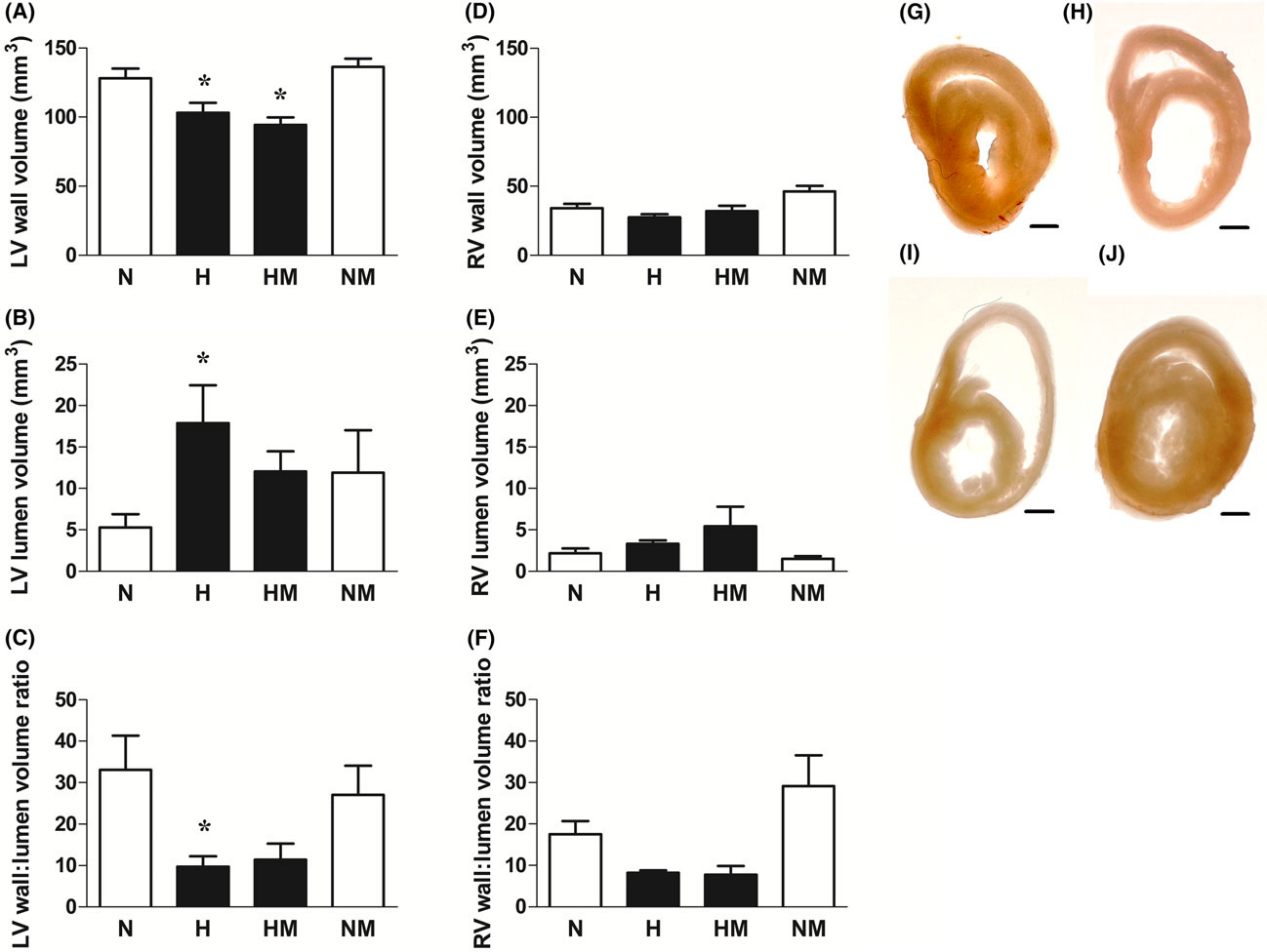
Cardiac stereology in normoxic or hypoxic chick embryos with or without melatonin treatment at day 19 of incubation. Values are mean ± S.E.M. at day 19 of the heart left ventricular (LV) wall volume (A), LV lumen volume (B), LV wall‐to‐lumen volume ratio (C), right ventricular (RV) wall volume (D), RV lumen volume (E), and RV wall‐to‐lumen volume ratio (F) of chick embryos incubated in either normoxia (N, n = 10), hypoxia (H, n = 10), hypoxia with melatonin (HM, n* =* 10), or normoxia with melatonin (NM, n = 10). Representative mid‐cardiac sections of day 19 chick embryos incubated in normoxia (G), hypoxia (H), hypoxia with melatonin (I), and normoxia with melatonin (J). Scale bar: 1 mm. Significant (*P* < 0.05) differences are as follows: * versus N. (one‐ or two‐way ANOVA with Tukey and Bonferroni post hoc test, respectively).

**Figure 7:**
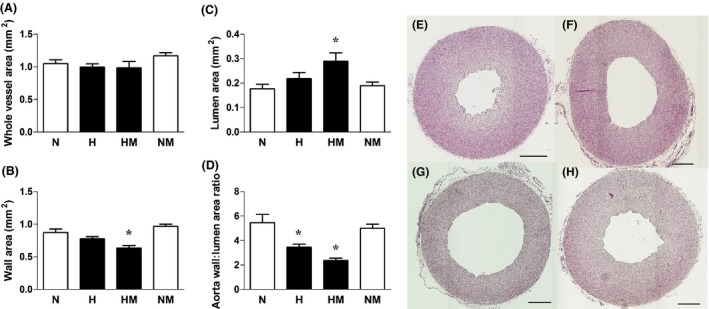
Aortic morphology in normoxic or hypoxic chick embryos with or without melatonin treatment at day 19 of incubation. Values are mean ± S.E.M. at day 19 of the total vessel area (A), wall area (B), lumen area (C), and wall‐to‐lumen area ratio (D) of the aorta of chick embryos incubated in either normoxia (N, n = 10), hypoxia (H, n = 10), hypoxia with melatonin (HM, n* =* 10), or normoxia with melatonin (NM, n = 10). Representative cross‐section of the aorta of day 19 chick embryos incubated in normoxia (E), hypoxia (F), hypoxia with melatonin (G), and normoxia with melatonin (H). Scale bar: 200 *μ*m. Significant (*P* < 0.05) differences are as follows: * versus N. (one‐ or two‐way ANOVA with Tukey and Bonferroni post hoc test, respectively).

Relative to normoxic embryos, embryos incubated under hypoxic conditions showed significantly reduced values for the wall volume and significantly increased values for the lumen volume of the left ventricle without affecting these values in the RV. Consequently, the ratio of the left ventricular wall to lumen volume was markedly decreased in hypoxic relative to normoxic embryos (Fig. 6A–J). The increase in left ventricular lumen volume and the reduction in the ratio of the left ventricular wall to lumen volume were no longer significant between normoxic embryos and hypoxic embryos treated with melatonin. However, melatonin treatment in hypoxic embryos did not significantly alter the reduced wall volume of the left ventricle (Fig. 6A–J). Melatonin treatment in normoxic embryos did not affect left or right ventricular morphology (Fig. 6A–J). Relative to normoxic embryos, embryos incubated under hypoxic conditions showed a reduced ratio of the wall to lumen area of the aorta (Fig. 7D). Melatonin treatment in hypoxic embryos increased the lumen area and decreased the wall area of the aorta, such that the reduction in the wall to lumen area of the aorta measured in hypoxic embryos was not significantly affected (Fig. 7A–D). Melatonin treatment in normoxic embryos did not affect the whole cross‐sectional area of the aorta (Fig. 7A–D).

**Figure 8:**
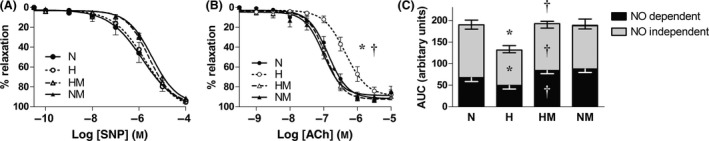
Peripheral vasodilator function in normoxic or hypoxic chick embryos with or without melatonin treatment at day 19 of incubation. Values are mean ± S.E.M. for relaxant responses to sodium nitroprusside (SNP, A) and to acetylcholine (ACh, B), and vasodilatation to ACh expressed as area under the curve before and after LNAME treatment (AUC, C) for femoral arterial segments isolated from chick embryos incubated in either normoxia (N, n = 10), hypoxia (H, n = 10), hypoxia with melatonin (HM, n* =* 10), or normoxia with melatonin (NM, n = 10). For (C), the AUC is presented for ACh‐induced relaxation (complete bar with positive S.E.M.), for ACh‐induced relaxation following treatment with L‐NAME (NO‐independent component, gray bar with negative S.E.M.), and for the remaining AUC after ACh with L‐NAME (NO‐dependent component, black bar with negative S.E.M.). Significant (*P* < 0.05) differences are as follows: * versus N. † H versus HM for pD2 (B) and AUC (C) (one‐ or two‐way ANOVA with Tukey and Bonferroni post hoc test, respectively).

The femoral vasodilator response to SNP was not significantly affected by hypoxic incubation or by treatment with melatonin under normoxic or hypoxic conditions (Fig. 8A). However, relative to normoxic embryos, embryos incubated under hypoxic conditions showed significantly impaired femoral vasodilator sensitivity to ACh, primarily by affecting NO‐independent mechanisms (Fig. 8B,C). Melatonin treatment in hypoxic embryos restored the femoral dilator response to control values by significantly enhancing both NO‐dependent and NO‐independent mechanisms (Fig. 8B,C). Melatonin treatment in normoxic embryos did not affect the femoral dilator reactivity to ACh (Fig. 8B,C).

The femoral vasoconstrictor responses to potassium and to phenylephrine were also tested. Relative to normoxic embryos, embryos incubated under hypoxic conditions had a significantly enhanced maximal constrictor response to potassium, while the responses to phenylephrine were similar. Melatonin treatment in hypoxic embryos did not affect the constrictor response to potassium, but it significantly increased the constrictor response to phenylephrine. Melatonin treatment in normoxic embryos did not affect the femoral constrictor reactivity to potassium or to phenylephrine (Potassium: N:2.0 ± 0.3; H:3.9 ± 0.7*; HM:3.9 ± 0.5*; NM:2.7 ± 0.1 mN/mm/*μ*m/1000. Phenylephrine: N:116 ± 5%; H:120 ± 5%; HM:156 ± 17%*; NM:123 ± 6% K^+^125 mm).

## Discussion

The data in this study show that treatment of the chronically hypoxic chick embryo with melatonin from day 13 of incubation, equivalent to *ca*. 25 wk of gestation in human pregnancy, rescues cardiac systolic dysfunction, impaired cardiac contractility and relaxability, increased cardiac sympathetic dominance, and endothelial dysfunction in peripheral circulations. The mechanisms mediating the protective effects on the cardiovascular system of melatonin in the chronically hypoxic chick embryo include reduced oxidative stress, enhanced antioxidant capacity and restored VEGF expression, and NO bioavailability. Therefore, the data in this study support the hypothesis tested that melatonin has direct protective effects on the fetal heart as well as the fetal vasculature in adverse development in addition to beneficial effects on placental function. As antioxidant‐induced protection in the chronically hypoxic chick embryo occurred despite administration after 13 days of hypoxic incubation, melatonin may be a plausible candidate for translation to human clinical therapy to rescue fetal cardiovascular dysfunction once fetal growth restriction (FGR) diagnosis has been possible.

In the present study, hypoxic development in the chick embryo increased hematocrit and cardiac VEGF expression, and it induced asymmetric growth restriction and affected cardiac and peripheral vascular function with consequences for the morphology of the heart and major vessels, such as the aorta. Hypoxia induces the activation of the hypoxia‐inducible factor‐1 (HIF‐1), which binds to the hypoxia response elements of the erythropoietin 26 and *vegf* 27, 28 genes, enhancing their transcription. Therefore, the data confirm that exposure of the chick embryo to 14% oxygenation from the beginning of incubation induced established molecular and hematologic indices of chronic fetal hypoxia.

The effects of chronic hypoxia from the beginning of incubation on the developing cardiovascular system are consistent with those described by other studies in the chick embryo and in other species. Several studies have reported peripheral vascular dysfunction, myocardial wall thinning, reduced myocardial contractility, and impaired cardiac systolic function following early developmental hypoxia in the rodent fetus and neonate, the chick embryos, and in young children 29, 30, 31. Sharma et al. 32 reported dilated cardiomyopathy, a lower maximal ventricular developed pressure with enhanced end‐systolic volume and decreased ejection fraction, consistent with impaired systolic function following chronic hypoxia in the chick embryo. Gilbert 33 reported that myocardial cell contractile function was impaired in the chronically hypoxic ovine fetus. Under these conditions, cardiac sympathetic dominance may be an adaptive response to maintain cardiac output. Accordingly, Lindgren and Altimiras 34 reported that chronic hypoxia sensitizes beta‐adrenergic receptors in the chick embryo heart. Of interest, sustained increases in myocardial contractility due to heightened sympathetic excitation have been strongly associated with future cardiovascular dysfunction and eventual heart failure in humans 35, 36. Therefore, the present study adds new conceptual insight into the literature, reporting that similar cardiac and vascular dysfunction triggered by early developmental hypoxia in the chick embryo can be diminished by melatonin treatment.

In the present study, melatonin treatment prevented the increased expression of molecular indices of oxidative stress in the chick embryo heart, such as nitrotyrosine, a footprint for peroxynitrite, and 4‐hydroxy‐2‐nonenal, an established marker of lipid peroxidation. Melatonin treatment also restored hypoxia‐induced decreases in the activity of the antioxidant catalase, and it increased the expression of GPx in the chick embryo heart. Diminished oxidant stress coupled with maintained or enhanced antioxidant defences are mechanisms which likely explain the capacity of melatonin treatment to restore cardiac NO bioavailability. Therefore, findings in the present study are not only consistent with developmental hypoxia triggering cardiovascular dysfunction secondary to oxidative stress 8, 10, 37 and with the protective effects of melatonin on the developing cardiovascular system being due to melatonin having antioxidant actions 38, 39, 40, 41, 42, but they further expand our understanding by showing that oxidative stress‐induced cardiac dysfunction triggered by developmental hypoxia can be rescued by antioxidant treatment starting much later than the onset of hypoxia. This is important in terms of translation to the human clinical situation, when chronic fetal hypoxia leading to IUGR can only be reliably diagnosed at around 25 wk of gestation.

We and others have reported that the antioxidant actions of melatonin increase umbilical blood flow via NO‐dependent mechanisms and that the indole amine is protective on placental function 14, 42, 43, 44. Consequently, maternal supplementation with melatonin in undernourished pregnancy in rats increased birthweight 13. Such findings have led to the design and implementation of a human clinical trial to test the therapeutic efficacy of maternal oral melatonin administration in protecting FGR 16. The lack of a protective effect of melatonin on FGR in the hypoxic chick in the present study is consistent with the beneficial effects of melatonin on growth being at the level of the placenta and therefore absent in avian species.

An elegant study by Tintu et al. 30 suggested that the mechanism of adverse actions of developmental hypoxia on the cardiovascular system included those mediated by VEGF, as systemic administration of recombinant VEGF during chick embryo development mimicked the hypoxia‐induced cardiac dilatation 30. Interestingly, in vivo treatment with sFlt‐1, a soluble receptor and a scavenger of VEGF, during hypoxic development protected cardiomyocyte contractility and prevented ventricular dilatation. Data in the present study support that VEGF is an involved mechanism triggering dilated cardiomyopathy in the hypoxic chick embryo, as melatonin treatment of the chronically hypoxic chick embryo normalized cardiac VEGF expression. Melatonin may restore the appropriate level of VEGF in the fetal heart by destabilizing the hypoxia‐induced HIF‐1*α* activity on the transcription of *vegf*, as reported in cancer cells 45.

It is of interest that the direct protective actions of melatonin on the developing cardiovascular system of the hypoxic chick embryo occurred at doses lower than those used to avoid jet lag in humans and achieving circulating concentrations similar to those measured in other studies 13, 14, 46. In control embryos, circulating melatonin levels peaked within 30 min of administration, and detectable levels of melatonin were still present 8 hr after treatment. It is known that genomic antioxidant actions of melatonin remain long after peak concentrations have been achieved 39, 47. In addition, the breakdown products of melatonin also have direct antioxidant actions 48. Combined, these findings suggest that daily treatment in the present study provided sufficient antioxidant cover. Interestingly, the profile of the change in melatonin concentration following administration between normoxic and hypoxic chick embryos was different, despite no difference in the total circulating concentration of melatonin achieved within the 24‐hr day between groups. The reason for the delay in the increase in melatonin concentrations following administration in hypoxic chick embryos is not clear, but it could be due to either a slower uptake of melatonin from the chorioallantoic vessels and/or faster metabolism and breakdown of melatonin in the normoxic embryo. A previous study in rats 49 supports both options as compared to normoxic controls, there was also a lag in the appearance of the peak plasma concentration and in the subsequent clearance of acetaaminophen in hypoxic rats following oral administration.

One final consideration is that a very recent study from our laboratory has reported that melatonin treatment in late gestation fetal sheep may weaken the fetal brain sparing circulatory response to an acute hypoxic episode as may occur intrapartum during normal labor 50. However, in that study only the effects of melatonin on peripheral rather than cerebral blood flow in the fetus were determined. Clearly, the beneficial effects of melatonin in protecting the developing fetal cardiovascular system in pregnancy complicated by chronic fetal hypoxia have to be balanced against the possible effects of melatonin adversely affecting the fetal brain sparing defence to intrapartum hypoxia. Therefore, there is an urgent need to establish the effects of melatonin on cerebral perfusion in the fetus exposed to acute or to chronic hypoxia, prior to translation to the human clinical situation.

In conclusion, melatonin has direct protective effects on the fetal heart and circulation in addition to beneficial effects on placental function in development complicated by chronic fetal hypoxia. We also provide the first evidence to support that melatonin can rescue cardiac dysfunction triggered by developmental hypoxia despite antioxidant treatment starting much later than the onset of hypoxia. Therefore, melatonin may be an attractive antioxidant candidate for translation to human therapy as it is not only able to be administered in tolerable doses but it also rescues fetal cardiovascular dysfunction long after the onset of chronic fetal hypoxia, thereby permitting antioxidant treatment following FGR diagnosis. Nevertheless, future research will have to balance these strong protective effects of melatonin on the developing cardiovascular system during chronic hypoxia against possible adverse effects of antioxidants on the fetal cardiovascular defence to acute hypoxia.

## Funding sources

Supported by the British Heart Foundation. Dino Giussani is the Professor of Cardiovascular Developmental Physiology & Medicine at the Department of Physiology Development & Neuroscience at the University of Cambridge, Professorial Fellow and Director of Studies in Medicine at Gonville & Caius College, a Lister Institute Fellow and a Royal Society Wolfson Research Merit Award Holder.

## Disclosures

The authors confirm that there are no conflict of interests.
